# Brain mechanisms underlying self-other distinction for bodily self-recognition

**DOI:** 10.3389/fncir.2026.1781653

**Published:** 2026-03-04

**Authors:** Yuuki G. Oka, Masaki Isoda

**Affiliations:** 1 Division of Behavioral Development, Department of System Neuroscience, National Institute for Physiological Sciences, National Institutes of Natural Sciences, Okazaki, Aichi, Japan; 2 Physiological Sciences Program, Graduate Institute for Advanced Studies, SOKENDAI, Hayama, Kanagawa, Japan

**Keywords:** brain, latent variables, self-other distinction, self-other inference model, self-recognition, state-space point-process model

## Abstract

Accumulating evidence indicates that single neurons in the primate brain specifically encode sensorimotor experience about the self or others. Although the self-other distinction has been a major focus of social neuroscience research, little is known about the underlying mechanisms that enable the recognition of bodily self. Here, we review the literature demonstrating that pre-reflective bodily self-recognition can be achieved through the spatiotemporally contingent integration of visual, somatosensory, and motor signals arising from sensorimotor experience. We propose a self-other inference model as a neural computation for self-other distinction, in which the likelihood of being oneself is updated constantly based on Bayesian causal inference using appearance, contingency, and perspective cues. The results of simulation incorporating a state-space point-process model revealed that our self-other inference model successfully captures the latent state representation about the self-other distinction from synthetic neural activity. We hypothesize that the self-other inference model is implemented by distinct brain areas that process individual cues and their integrative hubs. This hypothesis is experimentally testable using cutting-edge technologies such as area-specific or pathway-selective silencing.

## Introduction

The self is a multidimensional construct comprising a complex array of psychological, social, and physical components (James, 1890). It is inherently socio-psychological, as it develops through continuous interactions with other individuals, and is profoundly influenced by psychological processes such as perceived role expectations and social comparisons (Festinger, 1954; Goffman, 1959). Consequently, the self can manifest in context-dependent forms that vary across the conditions in which one functions, for example, as a professional, family member, or friend.

The socio-psychological self is anchored in the physical world and is shaped by various bodily sensorimotor experiences. Active motor engagement with the environment, from grasping an object to navigating a space, defines the self as an agent with boundaries and capabilities for intentional action. This active engagement is fundamental to developing a sense of agency (Gallagher, 2000; Haggard et al., 2002), which is the feeling of being the author and controller of one’s own actions and their consequences. Furthermore, the continuous sensory feedback generated by these movements contributes to the sense of body ownership (Botvinick and Cohen, 1998; Gallagher, 2000), establishing the body as uniquely and fundamentally “mine.”

The senses of agency and body ownership, collectively called the minimal self-consciousness or “minimal self,” are thought to play a role in pre-reflective bodily self-awareness (Gallagher, 2000). They are critical for integrating our social identity with our physical being, providing a coherent, lived experience of the self. They allow the self to transition from an abstract social entity to a unified identity that is grounded in concrete actions and capable of purposeful engagement with the world. Although the sense of self is a foundation for how we interact with the social world, its neural bases are unknown.

In this article, we first review empirical research into the pre-reflective bodily self in single-subject laboratory conditions. We then show that neural activity originally thought to be linked exclusively to one’s own sensorimotor experience can also be others-related. We argue that the identification of neural correlates genuinely associated with self-sensorimotor experience requires experimental conditions in which others are present. Then, driven by a Bayesian causal inference model that accounts for the sense of body ownership, we propose a self-other inference (SOI) model as a neural mechanism underlying self-other distinction, in which the moment-to-moment likelihood of being oneself is computed using appearance, contingency, and perspective information as critical cues. Furthermore, we show results of our simulation that the SOI model, combined with a state-space point-process model, successfully captures the latent state representation about the self-other distinction using synthetic neural activity. Finally, we discuss how the SOI model is implemented in the physiological brain and can be relevant to clinical conditions.

## Neural correlates of the pre-reflective bodily self in single-subject conditions

Experimental work on the neural correlates of the pre-reflective bodily self has been performed in the context of the sense of agency and body ownership. First, research into the sense of agency was facilitated in humans owing to the discovery of a phenomenon called intentional binding, i.e., a subjective temporal compression between a voluntary action and its sensory consequences (Haggard et al., 2002). The intentional binding effect is significantly attenuated during inactivation of the pre-supplementary motor area, but not the primary motor cortex (Moore et al., 2010), suggesting a role for the pre-supplementary motor area in the sense of agency. This observation accords with earlier findings that the pre-supplementary motor area is involved in attending to intention (Lau et al., 2004) and feeling a conscious urge to act (Fried et al., 1991). Another line of research showed that activation of the human parietal cortex evokes a conscious experience of wanting to move one’s own body part without concomitant muscular activity (Desmurget et al., 2009).

Second, a rubber hand illusion paradigm has been employed to investigate the sense of body ownership. In this paradigm, when human participants see a visible rubber hand being stroked while their own hidden hand is stroked simultaneously, they begin to perceive the rubber hand as their own (Botvinick and Cohen, 1998). The rubber hand illusion occurs when multisensory stimuli are experienced with plausible spatiotemporal patterns. Single neurons in parietal area 5 of monkeys encode not only static arm position derived from proprioceptive input but also the position of a false arm derived from visual input if the false arm looks plausibly like the real arm (Graziano et al., 2000). Notably, this visual response emerges immediately after the experimenter strokes the real and false hands synchronously, but disappears quickly when the strokes occur asynchronously (Graziano et al., 2000).

Although not examined directly in the context of pre-reflective self-recognition, neurophysiological recordings from cortical motor areas in macaques identified single neurons whose activity is increased preceding one’s own actions. A subset of these neurons shows greater responses before internally determined actions than before externally triggered ones (Okano and Tanji, 1987; Mushiake et al., 1991). Moreover, a sizable number of neurons in the cingulate motor areas start firing long before action initiation (Shima et al., 1991) in a similar time course to the readiness potential in humans (Kornhuber and Deecke, 1965). These findings led researchers to suggest that activity in cortical motor areas reflects aspects of the volitional control of “self-action.” However, as detailed in the next section, subsequent studies incorporating other individuals into the experimental environment revealed that such interpretations are not always valid.

## Redefining self-related brain activity in two-subject conditions

The addition of another subject to the existing experimental environment, thereby creating a minimal social context, revealed that the neurons active during one’s own sensorimotor experience can also be active during others’ sensorimotor experience. A prime example is the discovery of mirror neurons (di Pellegrino et al., 1992; Rizzolatti and Craighero, 2004), which fire when an individual performs a specific action, but also fire similarly when the individual merely observes another individual performing the same action. Such mirror properties can only be found by providing conditions in which one subject observes another undergoing a similar sensorimotor experience. An important implication of this discovery is that in order to test whether the given neural activity is genuinely associated with one’s own sensorimotor experience, it is essential to have others present.

Subsequent experiments using two monkeys positioned face-to-face provided a more nuanced picture. These studies demonstrated that the cerebral cortices and subcortical structures largely contain three types of neurons that are associated with bodily motor experience: self-neurons, mirror-neurons, and other-neurons (Yoshida et al., 2011; Báez-Mendoza et al., 2013; Grabenhorst et al., 2019; Ninomiya et al., 2020; Isoda, 2021). Interestingly, even the activity of self-neurons can change depending on the social relevance of others. Specifically, their activity is affected by whether their task partner is a physically present conspecific, a filmed conspecific, or a filmed object (Ninomiya et al., 2020; Ninomiya and Isoda, 2024). These findings are in line with the idea that self-recognition is shaped by social contexts. Paradoxically, understanding the neural basis for bodily self-recognition requires moving beyond the single-subject paradigm. This claim holds true not only in the context of motor experience but also in the context of sensory experience (Ishida et al., 2010). Even intentional binding is not confined to self-actions; it can also occur during joint actions (Strother et al., 2010; Jenkins et al., 2021).

Two critical questions are unsolved. The first concerns what aspects of pre-reflective self-recognition, i.e., the sense of agency or body ownership, the activity of self-neurons might be associated with. This question remains unanswered in part because of the limitations of the behavioral paradigms that can be used to examine the subjective recognition of self in non-human primates. Macaque monkeys are capable of passing a mirror self-recognition test, e.g., they touch a mark placed on their body only visible to them in a mirror (Chang et al., 2015, 2017), indicating that they recognize the reflection as themselves. This paradigm should enable researchers to shed light on how the activity of self-neurons is associated with pre-reflective bodily self-recognition.

The second question concerns how the brain might distinguish self from others. Although there are several frameworks that account for the mechanisms underlying the self-other distinction (Blakemore et al., 1999; Frith et al., 2000; Chang, 2013), they do not fully address its context-dependent and time-varying nature. In the next section, driven by the existing Bayesian causal inference model, we propose a model whereby individuals can determine whether a certain sensorimotor experience belongs to the self or another. We then discuss how this model can be implemented in the biological brain.

## SOI model for context-dependent, time-varying bodily self-recognition

The Bayesian causal inference model (Körding et al., 2007) was originally developed to infer whether two sensory signals, e.g., visual and auditory, have a common or independent cause. The core computation is to estimate the posterior probability, given sensory inputs, of the underlying hidden causal structures that cannot be observed directly. Beyond this multisensory perception, the Bayesian causal inference model has been extended and adapted to many other conditions including the sense of body ownership (Kilteni et al., 2015; Shams and Beierholm, 2022). Indeed, under this framework, neural activity in the monkey premotor cortex reflects the likelihood of integrating multiple sensory inputs (Fang et al., 2019; Qi et al., 2022). We conjecture that the Bayesian causal inference model can be applied further to the self-other distinction by considering whether multiple sensorimotor signals can be ascribed to the self or other.

We hypothesize that some of the contributing factors proposed for the sense of ownership (Kilteni et al., 2015) also have a fundamental role in the self-other distinction. These factors include appearance, multisensory contingency, and perspective. “Appearance” refers to morphological features, such as the shape and contour of the body part and the texture and color of the body surface. “Multisensory contingency” is associated with the sense of agency; when visual, tactile, and proprioceptive inputs arise exactly as predicted from motor output, subjects are more likely to perceive that the ongoing sensorimotor experience is their own. “Perspective” refers to the viewpoint of the observer; due to the anatomical limitations in observing one’s own body, first-person and third-person perspectives increase the likelihood that the observed body belongs to oneself and another, respectively.

On the basis of the premise discussed above, we propose a hypothetical model to account for a pre-reflective distinction between the self and other during sensorimotor experience. In this SOI model, we assume that the brain maintains two opposing hypotheses for each agent, i.e., whether a given agent undergoing the current sensorimotor experience is self, C=self , or other, C=other . The brain then constantly updates the probabilities of both hypotheses, p(C={self,other}∣X) , using sensory evidence, *X*. To do so, the brain considers three factors, i.e., appearance, multisensory contingency, and perspective, and computes independent likelihoods of being the self or other, as formulated by p(Xapp∣C=self) , p(Xcon∣C=self) , and p(Xper∣C=self) , respectively, in the case of self likelihood. Here, the temporal coincidence detection mechanism is inherent in Xcon . After computing these feature-specific likelihoods, the brain integrates them into a joint likelihood, p(Xapp,Xcon,Xper∣C=self) . This likelihood is combined using Bayes’ rule with a prior belief, p(C=self) , which is the baseline expectation before obtaining sensory evidence, *X*, thereby generating the posterior probability p(C=self∣Xapp,Xcon,Xper). This posterior probability reflects a moment-to-moment estimate of whether the agent undergoing the current sensorimotor experience is the self.

Now we discuss how the SOI model might be computationally implemented in the brain. Such discussions are useful for considering the feasibility of the proposed model. To achieve this goal, we outline several important points. First, the above-mentioned likelihoods, prior belief, and posterior probability are all latent variables that cannot be observed directly. Second, population neural activity is assumed to reflect the latent representations of task-relevant variables, as demonstrated for movement plans (Churchland et al., 2012), decision variables (Roitman and Shadlen, 2002), and sensory evidence (Hanks et al., 2015). Third, we similarly assume that the latent variables associated with the self-other distinction are also represented by population neural activity. Finally, to link the conceptual SOI model with actual neural dynamics, we reconstruct neural activity using a state-space point-process (SSPP) model (Smith and Brown, 2003; Eden et al., 2004; Truccolo et al., 2005), which serves as a formal framework to bridge the unobservable latent state and the observable neural spiking data.

**Figure 1:**
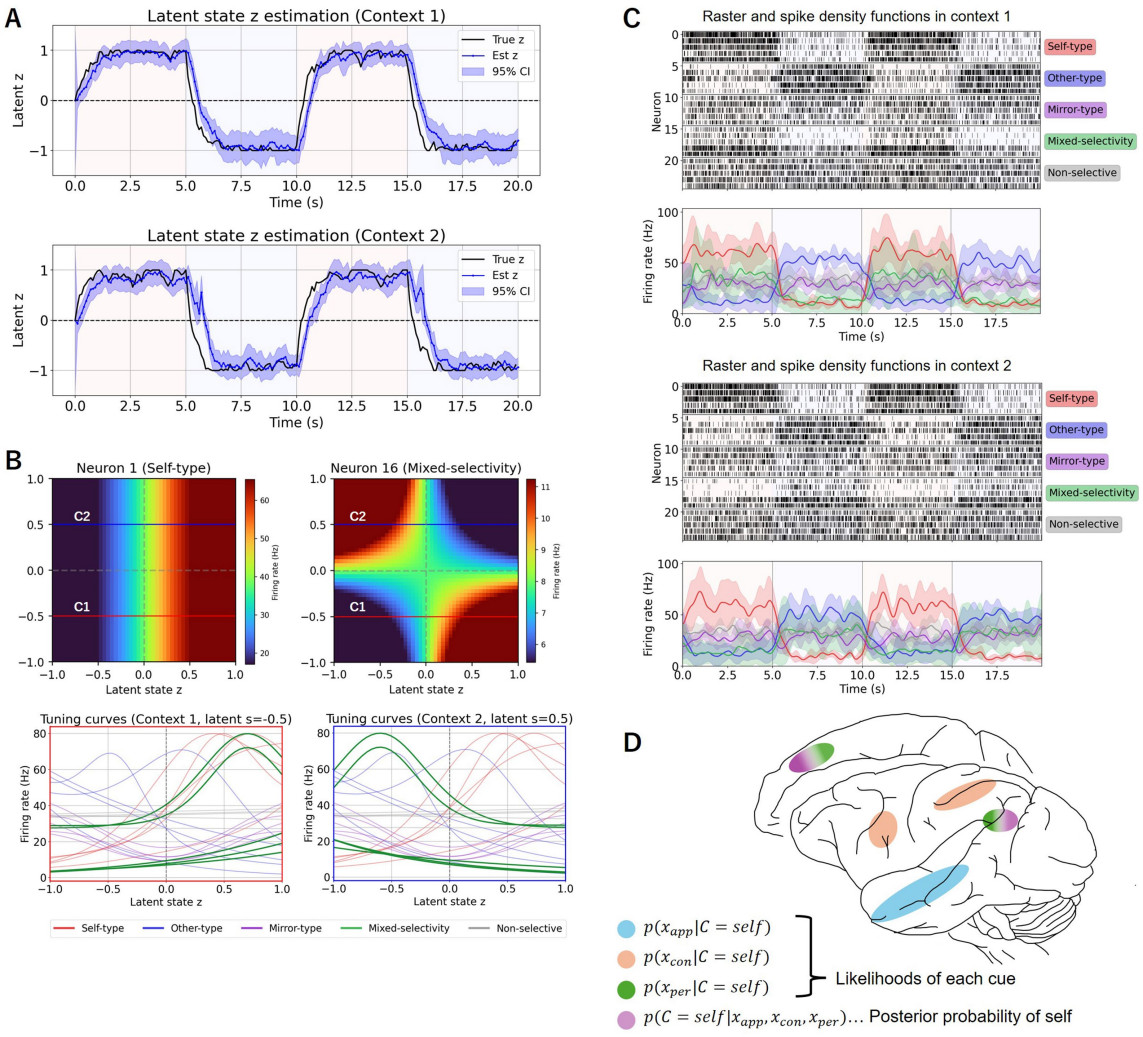
Schematic illustration of SOI model and simulation outcome. **(A)** An example of latent state dynamics for the self-other distinction. The image of either self-body or other-body is presented alternately every 5 s (corresponding to the light pink and light blue areas, respectively). True latent states are set arbitrarily (black line). Applying the SSPP model to the synthetic population neural activity in **(C)** yields estimated latent states (blue line) that reliably capture the true latent states. The model simulation was performed in 100 ms steps for computational efficiency; this granularity is independent of the actual temporal scale of neural computation in the brain. Positive and *s* negative values in the ordinate indicate the higher likelihood of the self and *s* other, respectively. **(B)** Tuning curves of the 25 neurons in **(C)**. Upper panels show two examples of neurons’ tuning to latent states z and s . The firing rate of a self-type neuron (left) monotonically changes depending on latent state z . The firing rate of a mixed-selectivity neuron (right) is determined depending on the combination of latent states z and s . Conditions in which latent state s is −0.5 (red horizontal lines, C1) and 0.5 (blue horizontal lines, C2) correspond to context 1 and context 2, respectively (bottom panels). Mixed-selectivity neurons are defined by their context-dependent activity. Note that the abscissa corresponds to the ordinate in **(A)**. **(C)** Simulated firing rate changes of 25 neurons with heterogeneous tuning curves to the self-other distinction. Some neurons increase firing as the latent state moves toward “self” (red), some toward “other” (blue), while others show no selectivity (gray). The same time step as in **(A)**. **(D)** Hypothetical brain areas representing the latent variables associated with the self-other distinction.

Here, we demonstrate the results of our model simulation. Suppose population activity is determined based on a dynamical latent state, $z_{t}$ , which is concerned with an internal belief about self versus other that fluctuates over time as sensory evidence changes (Figure 1A, black line). Given this latent state change, we assign distinct tuning curves to individual neurons for $z_{t}$ (Figure 1B), and generate synthetic neural activity (Figure 1C). Note that self-type neurons increase their firing as $z_{t}$ approaches “self” (Figure 1B, red lines), and other-type neurons increase their firing as $z_{t}$ approaches “other” (Figure 1B, blue lines). In addition to these self and other-selective neurons, we include mirror-type neurons that respond as $z_{t}$ approaches to either “self” or “other” (Figure 1B, purple lines), and mixed-selectivity neurons that change their tuning curves depending on task contexts (Figure 1B, green lines). The remaining neurons are nonresponsive (Figure 1B, gray lines). These response profiles reproduce the heterogeneous firing patterns seen in biological neurons. By using the generated neural activity and the SSPP model, we now obtain the inferred dynamics of the underlying latent state (estimated ${\hat{z}}_{t}$ ; Figure 1A, blue line). It is evident that the estimated latent state closely tracks the true latent state, demonstrating that population neural activity carries information that enables the decoding of low-dimensional representations of the self-other distinction.

## Discussion

We have presented the concept of an SOI model that was developed to account for how the brain might accomplish a distinction between the pre-reflective bodily self and others. The idea of the SOI model was driven by the Bayesian causal inference model (Körding et al., 2007) that now incorporates the sense of body ownership (Kilteni et al., 2015; Shams and Beierholm, 2022). We have demonstrated the potential to estimate the underlying latent representation of the self-other distinction from observable neural activity using the SOI model and the existing SSPP model. The implementation of the three factors and the posterior probability of being oneself into the SOI model seems to accord well with the known functional compartmentalization in the primate brain (Figure 1D). Specifically, the ventral vision pathway including the face patches (Tsao et al., 2006; Tsao and Livingstone, 2008; Freiwald and Tsao, 2010) is suitable for scrutinizing visual appearance. The intraparietal sulcus area and ventral premotor cortex may contribute to judging the multisensory contingency between visual, tactile, and proprioceptive inputs derived from one’s own movements (Graziano et al., 2000; Avillac et al., 2005; Petkova et al., 2011; Fang et al., 2019; Qi et al., 2022). The temporo-parietal junction and medial prefrontal cortex are involved in perspective taking (D’Argembeau et al., 2007; Mazzarella et al., 2013; Schurz et al., 2013; Martin et al., 2020; Ninomiya and Isoda, 2024), making them suitable for processing visual images from the first-person versus third-person perspectives. Finally, the temporo-parietal junction and medial prefrontal cortex have been implicated in representing the posterior probability of being the self as an integrated entity (Farrer and Frith, 2002; Northoff and Bermpohl, 2004; Moore et al., 2010; Igelström and Graziano, 2017). The SOI model suggests that silencing the activity in the integrative hubs, i.e., temporo-parietal junction and medial prefrontal cortex, is more likely to cause deficits in self-recognition across different task contexts, whereas silencing the activity in the face patches, intraparietal sulcus area, and ventral premotor complex, or their connections to the hubs, is more likely to cause domain-selective impairments of self-recognition.

The central tenet of our perspective lies in the functional distinction, or hierarchy, between these non-integrative regions and the integrative hubs: in the former, self-other distinction is achieved through domain-specific sensorimotor processes (such as appearance, contingency, or perspective), whereas in the latter, it emerges as a more abstract construct mediated by the integration of information across multiple sensorimotor domains. In this light, while these integrative hubs may also contribute to “reflective” self-other distinction in humans, our primary objective is to describe a fundamental mechanism that precedes linguistic ability and is applicable across species, including non-linguistic animals.

Several limitations of the present study should be noted. First, the successful decoding of latent states does not preclude other mechanisms by which the brain may implement self-other distinction. Our model provides a plausible framework, but it is not necessarily the exclusive one. Nevertheless, applying the SSPP model to recorded neural activity provides a powerful means to clarify whether the computational variables defined in the SOI model are indeed represented within the brain regions shown in Figure 1D. Second, the current model simplifies the dynamic process of temporal integration in contingency judgments. Specifically, the 100-ms simulation steps are a computational abstraction and do not reflect the actual temporal scale of neural computation. In biological systems, discriminating near-synchronous from asynchronous inputs may require nonlinear integration. In the brain, such processing can be achieved by multisensory neurons that exhibit nonlinear responses to the coincident arrival of sensory stimuli from different modalities. For example, a subset of neurons in the superior colliculus exhibits super-additive responses to temporally and spatially contingent multisensory inputs, exceeding the linear sum of their responses to each stimulus presented individually (Meredith and Stein, 1983; Alvarado et al., 2009). Although the precise mechanism underlying temporal coincidence detection remains to be fully elucidated, such neurons may provide important inputs to cortical areas involved in processing the contingency cue. Further refinement of our model could incorporate these nonlinear dynamics to more faithfully replicate physiological coincidence detection.

We expect that the SOI model can provide a useful framework to deepen our understanding of psychiatric and neurological disorders. For instance, delusions of control in schizophrenia can be considered a misattribution of self-generated actions and their consequences to external causes, most typically to others’ intentions (Frith et al., 2000; Blakemore et al., 2002). Difficulties with social interactions in autism spectrum disorder can be ascribed to atypical weighting or evidence integration in favor of others (Pellicano and Burr, 2012; Lawson et al., 2014). The symptoms in depersonalization disorder can be explained by disembodiment and lack of self-agency (Sierra and David, 2011). Thus, the SOI model may offer a computational vocabulary that helps us to understand diverse neuropsychiatric symptoms and provides a useful conceptual framework within a common latent inference problem.

## Data Availability

The original contributions presented in the study are included in the article/supplementary material, further inquiries can be directed to the corresponding author.
